# Concurrent Proinflammatory and Apoptotic Activity of a *Helicobacter pylori* Protein (HP986) Points to Its Role in Chronic Persistence

**DOI:** 10.1371/journal.pone.0022530

**Published:** 2011-07-15

**Authors:** Ayesha Alvi, Suhail A. Ansari, Nasreen Z. Ehtesham, Mohammed Rizwan, Savita Devi, Leonardo A. Sechi, Insaf A. Qureshi, Seyed E. Hasnain, Niyaz Ahmed

**Affiliations:** 1 Institute of Life Sciences, University of Hyderabad Campus, Gachibowli, Hyderabad, India; 2 Pathogen Biology Laboratory, Department of Biotechnology, School of Life Sciences, University of Hyderabad, Hyderabad, India; 3 National Institute of Nutrition, Hyderabad, India; 4 Department of Biomedical Sciences, University of Sassari, Sassari, Italy; 5 Department of Biotechnology, School of Life Sciences, University of Hyderabad, Hyderabad, India; 6 School of Biological Sciences, Indian Institute of Technology, Hauz Khas, New Delhi, India; 7 Institute of Biological Sciences, University of Malaya, Kuala Lumpur, Malaysia; Institute of Microbial Technology, India

## Abstract

*Helicobacter pylori* induces cytokine mediated changes in gastroduodenal pathophysiology, wherein, the activated macrophages at the sub-mucosal space play a central role in mounting innate immune response against the antigens. The bacterium gains niche through persistent inflammation and local immune-suppression causing peptic ulcer disease or chronic gastritis; the latter being a significant risk factor for the development of gastric adenocarcinoma. What favors persistence of *H. pylori* in the gastric niches is not clearly understood. We report detailed characterization of a functionally unknown gene (HP986), which was detected in patient isolates associated with peptic ulcer and gastric carcinoma. Expression and purification of recombinant HP986 (rHP986) revealed a novel, ∼29 kDa protein in biologically active form which associates with significant levels of humoral immune responses in diseased individuals (p<0.001). Also, it induced significant levels of TNF-α and Interleukin-8 in cultured human macrophages concurrent to the translocation of nuclear transcription factor-*κ*B (NF-*κ*B). Further, the rHP986 induced apoptosis of cultured macrophages through a Fas mediated pathway. Dissection of the underlying signaling mechanism revealed that rHP986 induces both TNFR1 and Fas expression to lead to apoptosis. We further demonstrated interaction of HP986 with TNFR1 through computational and experimental approaches. Independent proinflammatory and apoptotic responses triggered by rHP986 as shown in this study point to its role, possibly as a survival strategy to gain niche through inflammation and to counter the activated macrophages to avoid clearance.

## Introduction

Infection of human gastric mucosa with *H. pylori* is associated with different forms of gastro-duodenal diseases such as gastritis, peptic ulcers and gastric adenocarcinoma [1]. However, despite the fact that it colonizes more than 50% of the population worldwide, only a small subset of those infected develop more severe forms of gastric diseases; this may be due to various environmental and pathogen specific factors apart from different host immune responses [2], [3].

Establishment of successful colonization is a complex process that involves activities of several genome encoded virulence factors, aimed perhaps at survival through inflammation and defense *via* suppressing innate immune responses.

Once established in the host, *H. pylori* triggers activation of transcription factors and secretion of mucosal proinflammatory cytokines followed by cytoskeletal rearrangement, enhanced cell proliferation and apoptosis [4]. The induction of proinflammatory cytokines (IL-8 and IL-6) by *H. pylori* is mediated through NF-κB *via* recognition of toll-like receptors (TLRs) [5], [6]. Translocation of NF-κB by *H. pylori* promotes either the inflammatory process through induction of proinflammatory cytokines or regulates host defense by promoting or inhibiting apoptosis [7]. There are experimental evidences supporting the pro- and anti- apoptotic roles of NF-κB; its role in TNF-alpha /FasL mediated apoptosis has been described [8].

Given the proinflammatory responses aimed basically at gaining niche, the bacterium also appears to have evolved mechanisms to avenge primary defense maintained by the activated macrophages and lymphocytes [9]. This may involve selective inhibition of T-cell proliferation through up-regulation of Fas antigen [10], which is possibly mediated by cytokines (TNF-α and IL-1β), reactive oxygen metabolites and iNOS [11, 12, and 13]. This may reveal that although the persistent infection substantially increases mucosal inflammation, loss of activated macrophages proportionately limits clearance from the host [14], [15] leading to chronicity of inflammation.

*H. pylori* encodes several virulence associated molecules, including proapoptotic (such as VacA) [16] and anti apoptotic (such as CagA) [17] effectors and toxins, besides important virulence factors such as OipA, Ure, flagellins and adhesins. Although the functional coordinates of these factors have been extensively determined in different studies [18], [19], discrete associations of these with different disease outcomes have contradicting evidences [20]. In particular, microevolution and allelic diversity of the cagPAI, and vacA do not allow robust genotype-phenotype correlations thereby posing an obvious difficulty in linking the evolving virulence factors with the pathology [21]. In view of this, it is possible that the bacterium harnesses alternative strain specific factors [22] to achieve persistent infection. Also, there are several hypothetical and unknown proteins coded by *H. pylori* genome whose functional role in pathogenesis is unexplored. Therefore, it is pertinent to look into the biology of novel genes/proteins to get new insights into pathogenesis and phenotypic diversification of the bacterium in a changing host. The cache of many strain specific genes (the putative virulence factors) [23] comprises the ‘plasticity zone’ of *H. pylori* chromosome. Functional characterization of such genes and their involvement in pathogenesis of *H. pylori* could facilitate clear understanding of the development of peptic ulcer disease and gastric carcinoma. In this study, we describe efforts to systematically decipher the proinflammatory and apoptotic roles of one such putative virulence factor, HP986, and how this observation reinforces our understanding of the biology of *H. pylori* colonization and persistence.

## Results

### Association of HP986 with invasive disease outcomes and its distribution in clinical isolates

**Figure 1:**
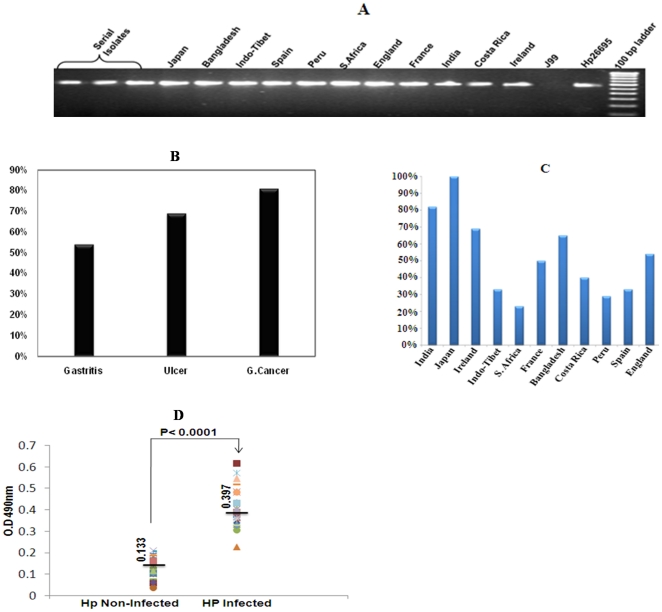
The locus hp986 was found to be associated with chronic gastric disease conditions. Panel A - Hp986 is widely distributed in different geographical regions; Panels B and C - Bar diagrams to represent % prevalence of hp986 in different disease categories and in different geographic regions, respectively; Panel D - Humoral responses directed against rHP986 were analyzed in sera collected from *H. pylori* infected patients belonging to different diseased categories and from control individuals (healthy controls and *H. pylori* non infected subjects). Antibody titers against rHP986 were compared between infected subjects and control individuals (*P*<0.001).

HP986 was found to be present in more than 61% of the total isolates we screened from many different geographical regions (Figure1 A, C). Overall, the presence of this gene was significantly associated with invasive disease (peptic ulcer and gastric carcinoma, 72%) outcomes (Figure 1B). This apparently contrasts previous observations [24] that describe HP986 to be gastritis specific. Also, the gene was found consistently conserved in all the three strains isolated from different niches of the stomach, almost a decade-apart [25], from a single patient (Figure 1 A). This suggests that despite a genome wide trend of extensive rearrangements in *H. pylori,* HP986 remains conserved.

### Protein sequence analysis and structure function prediction

**Figure 2:**
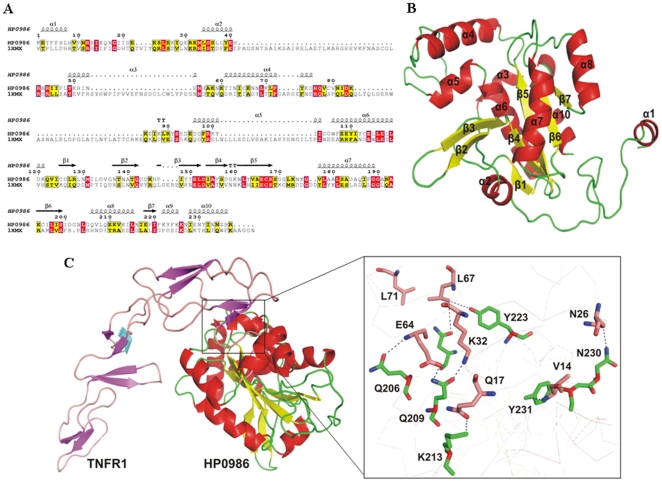
Sequence alignment and predicted 3D-structure of HP986. Panel A - Sequence alignment of HP986 with the hypothetical protein (VC1899) from *Vibrio cholerae* (PDB code 1XMX). Strictly conserved residues are highlighted in red and partially conserved residues are yellow. The sequence numbering refers to HP986. Final B - Predicted 3D-structure of HP986. The protein secondary structures elements are labelled and colored. Panel C - Interaction of HP0986 with TNFR1 using PatchDock and FireDock. The residues of HP986 and TNFR1 are colored in cyan and green respectively. The residues showing interaction between both proteins are labelled and displayed as stick model in element colors (carbon colored green/pink, nitrogen colored blue, and oxygen colored red). Hydrogen bonds are represented by black dashed lines.

HP986 was predicted to be *H. pylori* specific with no obvious sequence similarity in the available microbial sequence databases. Functional prediction showed high antigenic indices equivalent to ∼3.4 (DNAstar software, DNAStar Inc, USA). Due to the unavailability of crystallographic/solution structure of HP986, a search for possible homologs was carried out using several programs. Sequence-based search methods (BLASTp) did not provide any significant hit but sequence searches in PDB identified a template with 22% identity. This template (PDB ID: 1XMX) was a hypothetical protein, VC1899 from *V. cholerae* and a structural model of HP986 was built using it (Figure 2A). Total 50 solutions were obtained using Modeller9v8 [26] and solution No. 33 was considered the best among them on account of less energy. The quality of the structure was assessed using Ramachandran plot obtained *via* Procheck , which displayed 88.6% residues in most favored regions and 0.9% residues in disallowed regions. Consequently, ModLoop [27] was used to re-build the two residues in the modelled region followed by energy minimization. Results of the model validation using Procheck program were as follows: 89.0% residues in the most favored regions; 10.0% residues in the additional allowed regions; 0.9% residues in the generously allowed regions and 0.0% residues in the disallowed regions. Secondary structure analysis showed ten alpha helices and seven beta sheets in the modelled structure (Figure 2B). HEX [28], GRAMM-X [29] and PatchDock [30] programs were employed for unbound protein-protein docking with TNFR1 as receptor and HP986 as a ligand. Approximately 1000 predictions were generated using PatchDock and were submitted to FireDock [31] to refine 10 best solutions on the basis of global energy. Possible binding interface residues were identified using 3D2GO binding site prediction server [32]. Several of the lowest energy docking models emerging from this exercise placed the HP0986 on the side of the TNFR1. Among ten docked complexes, complexes 1 and 4 were identified as the plausible ones on the basis of minimum energy score and binding interface residues. A docking model of TNFR1-HP0986 is shown in Figure 2C, in which loop 1 and 2 regions of TNFR1 are docked onto α helices of HP0986.

### Expression and purification of rHP986

The over-expressed rHP986 was purified to homogeneity under native conditions as a His-tagged protein in *E. coli* BL21 (DE3). Homogeneity of the protein was further confirmed by fast performance liquid chromatography (FPLC). The purified protein upon fractionation on a 10% polyacrylamide gel showed a single band corresponding to ∼29 kDa on staining with Coomasie brilliant blue dye.

### Humoral responses to rHP986

A strong and significant humoral response (*p*<0.0001) was observed in *H. pylori* infected diseased subjects as compared to *H. pylori* negative individuals (Figure 1 D). Mean value of serum antibody levels (Mean ± SD) in *H. pylori* infected patients was 0.397±0.081 (Mean ± SD) as compared to *H. pylori* negative subjects 0.133±0051). However, rHP986 did not show disease stage wise (gastritis, peptic ulcer and gastric carcinoma) serum reactivity.

### rHP986 induces proinflammatory cytokines (TNF-α and IL-8) in a dose and time dependent manner

**Figure 3:**
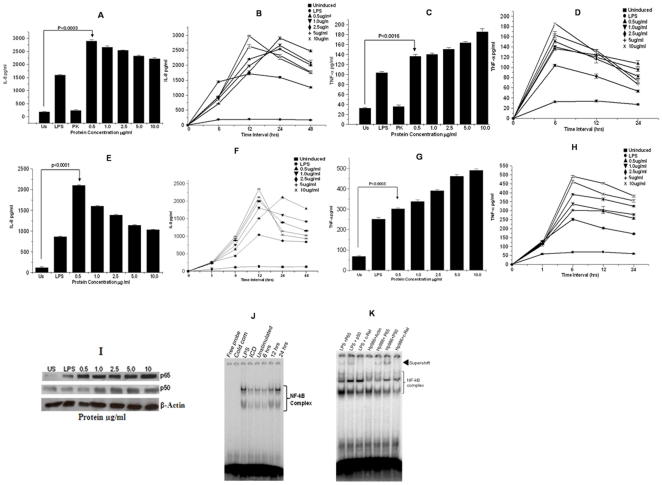
rHP986 stimulates the production of proinflammatory cytokines (IL-8 and TNF-α) through NF-κB. Panels A and B - Bar diagram and graph representing the amount of IL-8 secreted in Thp1-cells followed by exposure to rHP986; Panels C and D - The dose and time kinetics of TNF-α induction by rHP986 treated cells. Panels E and F - Dose and time dependent secretion of IL-8 by human PBMCs following rHP986 stimulation; Panels G and H – Dose and time dependent effect on the levels of TNF-α in human PBMC treated with rHP986. Results are shown as mean ± SE and represent findings from one of the three independent experiments. Panel I - Dose dependent effect of rHP986 on the translocation of NF-κB complex; lane1 - unstimulated cells, lane2 - cells treated with LPS, lanes 3 to 7 - cells treated with 0.5 µg/ml, 1.0 µg/ml, 2.5 µg/ml, 5.0 µg/ml and 10 µg/ml concentrations of rHP986 protein, respectively. Panel J - rHP986 mediated translocation of NF-κB complex was analyzed by electrophoretic mobility shift assay (EMSA); lane 1 – free probe, lane 2 – cold competition, lanes 3 and 4 – controls (LPS and ICD, respectively), lane 5 – negative control (cells without rHP986 treatment). Cells were treated with rHP986 (0.5 µg/ml) for varied time periods, lane6 - 6 hrs, lane7–12 hrs and lane8–24 hrs. Panel K - Supershift assay (K); specificity of rHP986 mediated activation of NF-κB complex was detected using specific antibodies against p65, p50 and c-Rel. Nuclear extracts prepared from differentiated cells treated with either rHP986 (lane5-p65, lane6-p50 and lane7-c-Rel) or LPS (lane1-p65, lane2-p50 and lane3-c-Rel) were incubated with antibodies as described in materials and methods. Nuclear extract from rHP986 treated cells incubated with β-actin was used as control - lane4.

rHP986 elicited strong cytokine response both in cultured / PMA differentiated Thp1 cells (Figure 3 A, C) and in human polymorphonuclear blood monocytes (PBMC) in a dose dependent manner (Figure 3 E, G). A significant increase in induction of TNF-α (p<0.0016) and IL-8 (p<0.0003) as compared to untreated cells was observed. Time kinetics revealed active production of TNF-α (P<0.0003) within 6 hrs of stimulation (Figure 3 D, H) which decreased slowly after 12 hours. In contrast, IL-8 secretion increased during this period with peak response noted at 12 hours post stimulation; levels were more or less maintained up to 48 hours (Figure 3 B, F). An unrelated His-tagged recombinant protein, isocitrate dehydrogenase (ICD) from *H. pylori* failed to demonstrate cytokine response even at the highest concentration of 10 µg/ml (data not shown). Further, inductions of these proinflammatory cytokines by rHP986 were not affected when the protein was treated with polymixin B. Additionally, proteinase-K treatment confirmed loss of rHP986 induced cytokine responses suggesting that the effect was due to rHP986 (Figure 3 A, C).

### rHP986 induces IL-8 through NF-κB

The role of transcription factor NF-κB in regulating the expression of IL-8 is already well established [33]. We observed a significant and proportionate increase in the activation of NF-κB complex in rHP986 treated cells in a dose (Figure 3 I) and time dependent manner (Figure 3 J) as compared to untreated cells and cells treated with LPS (Figure 3 I,J). Recombinant ICD from *H. pylori* was used as an unrelated control (Figure 3 J) and the levels of NF-κB complex corresponding to ICD were similar to those observed with untreated cells. Competition with unlabeled NF-κB DNA probe confirmed the specificity of the complex. Further, exclusive involvement of rHP986 in the activation of NF-κB complex was confirmed by using antibodies specific to p65, p50 and c-Rel. Addition of antibodies led to the supershift of p65 and p50 subunits in the extract of cells treated with rHP986 or LPS (Figure 3 K). No binding to anti- c-Rel antibody was observed (Figure 3 K). Actin was used as an equal loading control (Figure 3 K). It was thus confirmed that rHP986 up regulates NF-κB, which in turn induces IL-8 expression.

### HP986 functions through interaction with TNFR1

**Figure 4:**
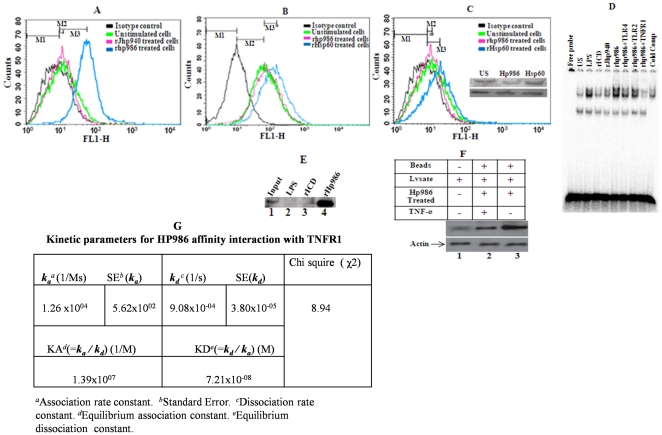
Interaction of HP986 with TNFR1 analyzed by flow-cytometry and immunoprecipitation. Panel A - Enhanced expression of TNFR1 following treatment with rHP986; Panel B - HP986 did not stimulate expression of TLR2 - levels were equal to the cells that had not received any protein treatment; Panel C - Treatment of HP986 had no visible effect on TLR4 expression [in the inset, lane 1 - untreated cells, lane 2 - cells treated with HP986 and lane 3 - cells treated with rHsp60 (from *M. tuberculosis*) used as control; Panel D - EMSA showing effects of neutralizing antibodies against TNFR1, TLR2 and TLR4 on the translocation of NF-κB complex in the cells stimulated with HP986 [lane1- free probe, lane2 – unstimulated/untreated cells, lane 3- cells treated with LPS, lane4 – cells treated with ICD, lane5- cells treated with JHP940, lane6 - cells treated with HP986, lane7- cells treated with HP986 and neutralizing antibody against TLR4, lane8 - cells treated with HP986 and neutralizing antibody against TLR2, lane9 - cells treated with rHP986 and neutralizing antibody against TNFR1 and lane 10 - specific competitor (unlabelled NF- κB consensus probe)]; Panel E -Immunoprecipitation assay showing interaction of rHP986 with TNFR1, lane 1 - input, lane 2 – cells treated and incubated with LPS, lane3 - cells treated and incubated with ICD, lane 4 - cells treated and incubated with rHP986. Immunoblot was developed using antibodies against TNFR1. Panel F - Role of endogenous TNF-α in binding to TNFR1 was ruled out using neutralizing antibodies against TNF-α; lane 1 - whole cell lysate of cells, lane 2 - cells incubated with rHP986 in the presence of neutralizing antibody to TNF-α, lane 3 - cells incubated with rHP986 in the absence of neutralizing antibody to TNF-α. Immunoprecipitate was pulled down using Talon beads as discussed in materials and methods section. Blot was developed using anti-TNFR1 antibody. Equal protein loading was confirmed by reprobing the blot with β-actin. Panel G – Summary of kinetic parameters for rHP986 affinity interaction with TNFR1: *a* =  Association rate constant, *b* =  Standard Error. *c* =  Dissociation rate constant *d* =  Equilibrium association constant *e = * Equilibrium dissociation constant.

rHP986 triggered the expression of TNFR1 by the Thp-1 differentiated macrophages (Figure 4 A). We also tested the possible interaction of rHP986 with TLR4 and TLR2; however, rHP986 treatment did not have any effect on toll-like receptor expression (Figure 4 B, C). This finding was further confirmed by analyzing the antagonist effect of TNFR1 receptor on NF-κB translocation. Pretreatment of cells with neutralizing antibodies against TLR4 and TLR2 did not abrogate NF-κB translocation (Figure 4 D), however, this did happen when cells were pretreated with a neutralizing antibody against TNFR1, suggesting the possible role of rHP986 in increased TNFR1 expression (Figure 4 D).

Interaction of rHP986 with TNFR1 was further validated by immunoprecipitation using anti-TNFR1 antibodies. The receptor was detected in the eluate treated with rHP986 but could not detect any corresponding signals in the cell lysate treated with either LPS or recombinant ICD (Figure 4 E). Exclusive interaction of rHP986 with TNFR1 was further confirmed in competition with neutralizing antibodies against TNF α; the amount of immune complex detected was less as compared to that seen in the absence of TNF-α (Figure 4 F). This suggested a direct interaction between TNFR1 and rHP986 and ruled out any possible role of endogenous TNF-α. Further, BIAcore® (Surface Plasmon Resonance) (GE Healthcare Ltd.) analysis provided insights into the interaction of rHP986 and TNFR1 interaction. Using 1∶1 Langmuir binding model to fit our binding curve, we found that rHP986 indeed binds with TNFR1 (*k_a_* = 1.26×10^4^±5.62×10^2^ Ms^−1^) on the biosensor surface. The resultant complex was found to be highly stable as illustrated by slow dissociation rate (*k_d_* = 9.08×10^−4^±3.80×10^−5^ s^−1^). A good binding fit (χ2 = 8.94) was obtained confirming the above values of *k_a_* and *k_d_* (Figure 4 G).

### rHP986 induces Fas mediated apoptosis

**Figure 5:**
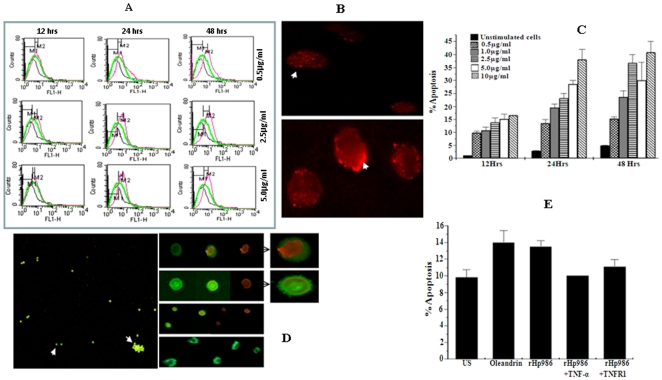
rHP986 induces Fas expression and apoptosis of cultured macrophages *via* a TNFR1 dependant pathway. Panel A - rHP986 induced Fas expression on the surface of differentiated macrophages as quantified by flow cytometry with anti-Fas antibody. Dose dependent increase in Fas expression in response to treatment with increasing concentration of rHP986 (0.5 µg/ml, 2.5 µg/ml and 5.0 µg/ml) observed across varied time intervals (12 hrs, 24 hrs and 48 hrs) is indicated by graph with pink line while the graphs with green line indicate response of cells without protein stimulation. Panel B - Enhanced Fas expression on cell surface after stimulation with rHP986 as compared to cells without recombinant protein stimulus. Panel C - Quantification of apoptosis in the cells treated with increasing concentration of rHP986 for varied time intervals. Results are represented as (mean±SE) percent apoptotic cells per 300 cells. Panel D - Morphological identification of apoptotic cells by acridine orange and ethidium bromide staining method. Arrow head indicates live cells with normal morphology (green fluorescence) and arrow shows cells that have undergone apoptosis after stimulation with rHP986. Black arrows indicate condensed marginal nucleus, apoptotic body formation and membrane blebbing. Panel E - Bar diagram showing inhibition of apoptosis when the cells were stimulated with rHP986 in the presence of neutralizing antibody against TNFR1 (rHP986+TNFR1) as compared to cells stimulated with only rHP986. Oleandrin was used as positive control; US - unstimulated cells. Results are shown as mean ± SE.

Considering the interaction of rHP986 with TNFR1, its possible involvement in inducing Fas expression was tested as the latter is known to function in synergy with TNFR1 and constitutively regulates downstream signaling cascade leading to apoptosis [11]. Pretreatment of PMA differentiated cells with rHP986 effectively regulated Fas expression in a time and dose dependent manner. Comparative expression analysis with increasing protein concentration at different time points revealed significant and proportionate increase in Fas expression up to 24 hours (Figure 5 A). Expression levels declined after 12 hrs in cells subjected to higher protein dose (5.0 µg/ml). This could be due to possible increased cell death. Immunocytochemical staining also showed an increased expression of Fas on the surface of rHP986 stimulated cells as compared to unstimulated ones (Figure 5 B).

Corroborating our findings with an earlier study [13] on the synergistic function of TNFR1 and Fas in inducing apoptosis, we evaluated the potential of rHP986 as an apoptosis-inducing agent. rHP986 triggered apoptosis in cultured macrophage cells in a dose and time dependent fashion (Figure 5 C). Furthermore, apoptosis as a function of Fas expression was also mechanistically shown through binding with annexin-V and acridine orange (Figure 5 D). A substantial and proportionate increase in cell death was observed when the cells were treated with increasing concentration of rHP986 (0.5 µg/ml-10 µg/ml) for varied time intervals; up to 48 hours (36.65%±3.25% to 41.0%±4.2%) as compared to untreated cells (10.65%±1.85%). This pro-apoptotic property of rHP986 declined significantly upon blocking its interaction with TNFR1 (Figure 5 E) indicating the involvement of rHP986 in TNFR1mediated cell death. Similar results were obtained when the cells were stimulated in the presence of neutralizing antibody against TNF-α, suggesting that the effect was not secondary to endogenous TNF-α. As expected, His-tagged ICD protein from *H. pylori* failed to induce apoptosis. A comparative analysis between oleandrin (a known inducer of apoptosis) [34] and rHP986 also confirmed the latter being an equally potent inducer of apoptosis (Figure 5 E). Collectively, all these findings confirm rHP986 to be a potent apoptosis-inducing agent.

## Discussion

Novel genes constantly emerge from newly sequenced replicate genomes. This paradigm was supported by the analyses wherein the pan-genome of a true bacterial species remained ‘open’ and each new genome sequence would identify dozens of new genes in the existing pan-genome of *Streptococcus agalactiae*, for example [35]. It is clear also from previous studies that a pool of strain specific genes in pathogens such as *H. pylori* termed the ‘plasticity region cluster’, could be useful in adaptation to a particular host population [36]. This pathogen shows a very strong geographic adaptation and is known for harboring up to 45% strain specific genes with most of them gained through horizontal gene transfers [36]. Recently, the members of the plasticity region cluster were shown to be likely involved in promoting proinflammatory potentials of some of the strains, possibly providing a survival advantage [37], [38].

However, a majority of the plasticity region genes /proteins are yet to be fully characterized. We recently reported functional characterization of JHP940, a novel antigen from this region that has shown potential proinflammatory activity [37]. However, it is not clear if plasticity region proteins provide any survival advantage to the pathogen and the mechanisms thereof. The present study attempted to explore functional aspects of HP986 as a putative virulence factor and to examine its prevalence in clinical isolates from different geographical regions. Also, we performed a series of activity experiments to elucidate its role in pathology; in particular, its proinflammatory and apoptotic activity in human macrophages. We used differentiated human macrophages since *H. pylori* considerably recruits and excites macrophages in the gastric submucosa to initiate a chronic and persistent trail of inflammatory activities leading to certain patho-physiological changes [38], [39].

In our observation, presence of HP986 gene was found to be significantly associated with invasive disease outcomes (ulcer and gastric cancer) as compared to gastritis (Figure 1 B); this contrasts a previous report [24] about its prevalence in strains linked to gastritis cases alone. In our study, low positivity was recorded in gastritis causing strains from Peru and South Africa (Figure 1 C). Moreover, the presence of HP986 was found to be independent of *cag*A and *vac*A status of the strains tested by us.

Despite traditionally high allelic diversity in *H. pylori*, HP986 was found to be evolutionarily conserved as observed for a period of ten years in strains isolated from different niches of the stomach of a single patient (Figure 1A) [25]. This suggests conserved maintenance of the locus in the genome thereby pointing to its essential role in pathobiology of *H. pylori*.

Given our theoretical observations on high antigenicity of HP986, *in silico* analysis showed several putative B-cell epitopes; we experimentally tested its ability to elicit humoral and cellular immune responses. Significant humoral immune responses induced by HP986 may be important in diagnostic development given the fact that many candidate antigens have suffered due to high genetic variability across different regions and cross reactivity with other related organisms [40]. Our analysis showed significantly high antibody titers in *H. pylori* infected invasive disease patients when compared with healthy controls or non-infected individuals (Figure 1D). This points to the extracellular abundance of HP986 protein and its role in stimulating immune response. Further, since the NF-κB activation in *H. pylori* is often type IV dependent, we can not rule out secretion of HP986 protein through a type IV secretary system. Alternatively, it is possible that the protein might be directly released into the extracellular space in the aftermath of autolysis [41], [42].

While considering the fact that *H. pylori* proteins released into extracellular space may find their way into the submucosa and augment proinflammatory signaling [39], we looked at rHP986 to potentially augment proinflammatory cytokine secretion from macrophages (IL-8 and TNF-α) consequent to NF-κB activation. These presumptions are consistent with an earlier study describing effect of *H. pylori* or its products on NF-κB (p65/p50) mediated transactivation of IL-8 [6]. Interestingly, our observed stimulation of IL-8 was found to be secondary to TNF-α secretion; maximal concentration was detected as early as 6 hrs (Figure 3 D, H) as compared to 12 hrs for IL-8 (Figure 3 B, F). The apparent decrease in TNF-α concentration may be due to the binding of TNF-α with the soluble TNF receptors. As a consequence, less TNF-α concentration was detected in the culture supernatant after 12 hours. Based on these findings, we propose the role of HP986 in cytokine mediated gastric injury in a similar way as shown previously for the airway epithelial inflammation triggered by *Staphylococus* protein-A [43]. The effect was consistent when tested in both Thp1 differentiated macrophages (Figure 3 A–D) and human PBMCs (Figure 3 E–H). It is known that *H. pylori* infection disrupts tight intracellular junctions and transports its products into the gastric submucosal space to augment infiltration of mononuclear cells [44]. This strategy perhaps helps the bacterium to establish persistent infection as it feeds on inflammatory exudates for carbon source derived from mucosal sugars and thereby gains a niche. Also, it inhibits expansion of antigen specific T-cells as a mechanism of immune evasion [15]. Studies support the notion that IL-8 activity attracts mononuclear cells, and TNF-α triggers Fas mediated apoptosis of activated macrophages [45], [46]. TNF-α works in an autocrine fashion by up regulating Fas expression on the surface of activated macrophages by binding with TNFR1 [13] and shares a hierarchy of downstream events (FADD) leading to apoptosis [14], [47]. Nevertheless, there are contradictory reports supporting pro and anti apoptotic roles of Fas in different cell lineages but antigen behavior also determines cell fate [48]. Our observed synergistic function of TNFR1 and Fas is consistent with the previous findings [46], [49]. Having identified the dependency of Fas on TNFR1, we anticipated that rHP986 actually binds to TNFR1 and thus mimics signalling through TNF-α. We confirmed this interaction through a series of immunological and biophysical measurement and our binding results were in accordance with computational modeling of HP986 and TNFR1 interaction; Also, we could not observe any binding with TLR4 or TLR2 (see results).

**Figure 6:**
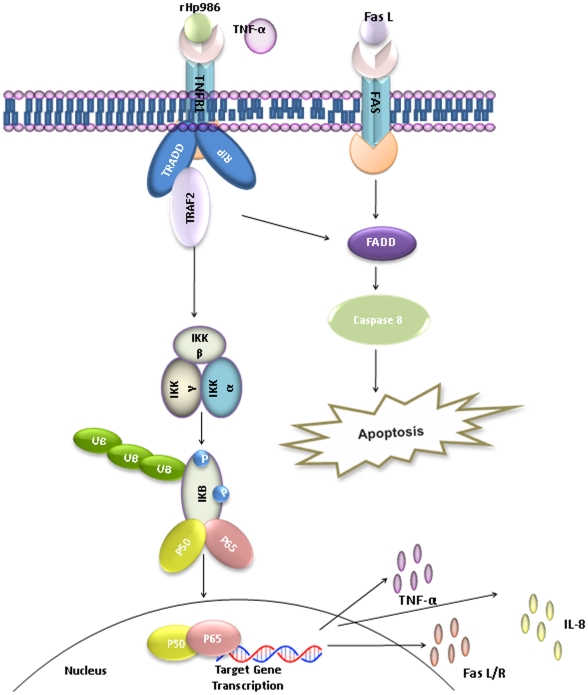
Schematic representation of major signaling pathways initiated following the binding of HP986 with TNFR1. The proposed interactions possibly activate downstream signaling cascades leading to macrophage apoptosis and induction of proinflammatory cytokines. TRADD: TNFR1 associated death domain, FADD: Fas- associated death domain, TRAF2: TNFR- associated factor 2, RIP: Receptor interacting protein.

Taken together, these observations allow us to propose a model of putative bacterial strategy (Figure 6) harnessed for survival and possibly for maintaining a balance between recruitment and activation of macrophages and their suppression by TNFR1 mediated apoptosis. This strategy potentially projects HP986 as an important player involved in both proinflammatory and apoptotic cascades. It is therefore highly probable that HP986 is a novel virulence factor and possibly an important effecter in gastritis and peptic ulcer disease and various other outcomes of chronic *H. pylori* infection such as gastric adenocarcinoma. While we do not know how many such virulence factors operate behinds the pathology triggered by this manipulative pathogen, understanding of each of them in depth is necessary to devise strategies to control progression of the infection towards more serious outcomes. Future efforts are indeed necessary to understand molecular structure of this protein to gain insights into intricacies of its function and how its role is regulated *in vivo*.

## Materials and Methods

### Ethics statement

The study was approved by the Institutional Biosafety Committee of the University of Hyderabad, India. Informed consents were obtained from all patients whose sera samples were used (all patients sampled were adults).

### Geographic distribution of the locus Hp986 and its genetic stability

Distribution of hp986 gene was analyzed in clinical isolates from different diseased subjects (gastritis n = 152, duodenal ulcers n = 68, gastric cancer n = 27) belonging to various geographical regions (India, Spain, South Africa, Japan, France, Peru, Ireland, England, Costa-Rica, Indo-Tibet and Bangladesh). PCR was performed using gene specific primers as described earlier [24].

### Computational modelling of HP986 protein-protein interactions

The 3D structure of query protein was predicted by automated homology modelling program, Modeller9v8 [26]. The protein template 3D structures used in the study were downloaded from RCSB Protein Data Bank (PDB). Amino acid sequences of HP986 were aligned with PDB ID 1XMX to derive the predicted secondary structure using the online tool, ESPript [50]. The geometry of model was checked with PROCHECK tool available with PDBsum program [51]. Molecular visualization and general analysis were done using the program PyMOL [52]. *In silico* docking experiments were performed using PatchDock [30] and then further refined and ranked with FireDock [31]. In crystal structure, the unliganded TNFR1 (PDB ID: 1NCF) exists as a dimer, and therefore only one molecule of TNFR1 (receptor) was taken for unbound protein-protein docking with HP986 model (ligand) under default complex-type settings.

### Cloning, expression and purification of HP986

The construct for recombinant protein expression was generated by cloning the PCR product spanning 711 bp at site *XhoI/HindIII* of pRSETA. The construct was then propagated into *E.coli* BL-21 (DE3) expression host and the over expressed his-tagged protein was purified by affinity chromatography (Ni^2+^-NTA, Qiagen) [53]. Further size exclusion chromatography was performed using Superose -1210/300 GL column (GE Healthcare Ltd.) in buffer containing 20 mM Tris-Cl and 300 mM NaCl pH 8.0. The recombinant protein was quantified using Bradford's reagent [54]. Also, purified rHP986 was treated with polymixin-B to remove possible endotoxin contamination.

### Analysis for humoral responses

A total of 70 human sera were collected after obtaining informed consents from different subjects having endoscopically proven gastritis, peptic ulcer and gastric carcinoma. Sera from subjects reported to be *H. pylori* negative (as ascertained by C^14^urea breath test) were used as negative control (n = 17). Humoral response against the rHP986 was determined by Enzyme linked immunosorbent assay (ELISA) as described previously [53]. Concentration of recombinant protein and sera was predetermined using serial dilutions to obtain optimum antibody titers (data not shown). Each ELISA experiment was repeated at least thrice with and without replicates.

### Cell culture experiments

Approximately 1×10^6^ human monocyte cells per well (Thp1) (ATCC, USA) were differentiation into adherent macrophage like phenotype using phorbol-12 myristate 13 acetate at a concentration of 5 ng/ml (Sigma, USA). These cells were induced using increasing concentration of rHP986 (0.5 µg, 1.0 µg, 2.5 µg, 5.0 µg, and 10 µg/ml) and incubated for varied time interval. Cells without protein stimulus (unstimulated cells) and the cells stimulated with proteinase K-treated rHP986 served as negative control. LPS (*E. coli*, Sigma) treated cells were used as positive control. A non relevant control was the His-tagged ICD from *H. pylori* purified under similar conditions. Culture medium collected at different time intervals (6 hrs, 12 hrs, 24 hrs and 48 hrs) was stored at −80°C until assayed.

### Polymorphonuclear blood monocyte cell (PBMC) culture

PBMCs were isolated from heparinized venous blood taken from a voluntary donor using ficoll-histopaque density gradient as described previously [55]. The cell viability was checked by trypan blue dye exclusion method and was found to be 90%. Approximately 0.5 million cells/well were seeded in 24 well plate in RPMI 1640 media supplemented with 10%FBS and 2 mM glutamine. Cells were treated with rHP986 protein as described above.

### Cytokine assay

Amount of IL-8 and TNF-α secreted in the culture medium was determined using commercially available optEIA ELISA Kit (BD Biosciences, USA) as per manufacturer's instruction. The cytokine levels were calculated using the recombinant standard provided within the kit.

### Cell extract preparation and Electrophoretic mobility shift assay

Cytoplasmic and nuclear extracts were prepared and translocation of NF-κB complex was determined by electrophoretic mobility shift assay (EMSA) as described earlier [56]. Unlabeled NF-κB consensus probe was used as specific competitor. For supershift assay 5-10 µg of rabbit polyclonal anti p65, p50, c-Rel antibodies (Santacruz Biotechnology, USA) were used. After electrophoresis the gel was dried and analyzed by autoradiography.

### Immunobloting

Immunobloting was performed as described previously [57]. Different antibodies such as those against p65, p50, c-Rel, TNFR1, TLR4 and TLR2 (Santacruz Biotechnology, USA) were used. Immunoreactive proteins were detected using enhanced chemiluminescence kit according to manufacturer's instructions (Amersham Inc., USA). β-Actin was used to confirm equal loading of the samples.

### rHP986 binding assay by flow cytometry

Binding of rHP986 with cell surface receptors such as TLR4, TLR2 (Imgenex, USA) and TNFR1(Santacruz Biotechnology, USA) was analyzed using specific antibodies by flow cytometry. FITC conjugated mouse IgG1 antibody (Santacruz Biotechnology, USA) was used as isotype-matched control Antibody. At least 10,000 cells were scanned per sample.

### Immunoprecipitation

0.1 mg/ml of cell extract was incubated with rHP986 overnight at 4°C and the immune complexes were trapped using Talon resin (Clontech, USA) or protein A/G agarose beads (Santa Cruz, USA). Immune complexes were separated on 10% SDS-PAGE and the immunoblot was developed using enhanced chemiluminescence kit (Amersham Inc, USA). Recombinant ICD from *H. pylori* and LPS were used as controls.

### Kinetic analysis of interaction using Surface Plasmon Resonance (SPR)

Binding kinetics of rHP986 with soluble humanTNFR1 (hu TNFR1) were analyzed using BIAcore® 3000 SPR system (GE Healthcare Ltd.). Human TNFR1/Fc (Sigma, USA) was immobilized by an amine-coupling method over a research grade CM5 sensor chip (BiAcore, Uppsala, Sweden) up to a resonance unit of 150. A reference surface was used as a blank to correct instrumental and buffer effects prior to protein injection. During the association phase, the purified rHP986 had been serially diluted in running buffer (PBS, BiAcore, Uppsala, Sweden) at 100 nM, 400 nM, 800 nM, and 1200 nM and were allowed to pass individually over the immobilized TNFR1 at a flow rate of 30 µl/min for 3 minutes. During the dissociation phase, PBS solution was applied to the sensor chip at a flow rate of 30 µl/min for 4 minutes. The sensor surface was regenerated between each binding reactions by two washes of 30 s each with 5 M NaOH as evaluated by baseline response. The data was analyzed with BIAEVALUATION 4.1 software (GE Healthcare Ltd.) using simple 1∶1 Langmuir interaction model.

### Assay of CD95 for rHP986 mediated apoptosis

Expression of CD95 was analyzed by flow cytometry using FITC conjugated anti-Fas monoclonal antibody (CD95, BD) [58]. FITC conjugated mouse IgG1 antibody (Santa Cruz) was used as isotype-matched control antibody. Cells stimulated in the presence of neutralizing antibody to TNFR1 (50 µg/ml) were used to check the involvement of TNFR1 in the regulation of Fas expression.

### Analysis of CD95 expression by Immunocytochemistry

Cells were sedimented on glass slide and fixed in 1% paraformaldehyde at 4°C for 30 minutes. For antigen staining cells were incubated with rabbit polyclonal anti-Fas antibody (1∶300, Santa Cruz, USA) for 1–2 hours at 37°C. Unbound antibody was washed off with PBS and the cells were further incubated with Alexafluor [44] conjugated anti-rabbit IgG (1∶200, Molecular probes, USA) for 30 minutes at room temperature [10]. Enhanced Fas expression was analyzed by fluorescence microscopy (Ziess epifluorescence microscope).

### Analysis of apoptosis induction by rHP986

Apoptosis assays were performed using acridine orange/ethidium bromide staining [11] and annexinV kit method (BD pharmingen, USA). For acridine orange and ethidium bromide assay, approximately 100 cells were counted in three randomly selected fields and the rate of apoptosis was expressed as mean percentage of total 300 cells counted. For annexinV, percentages of apoptotic cells were expressed as total % of annexinV^+^ and PI^+^ cells after subtracting background fluorescence [46]. Cells treated with rHP986 in presence and absence of neutralizing antibodies to TNF-α and TNFR1 were used to determine the role of rHP986 in the induction of apoptosis. Oleandrin, a known potent inducer of apoptosis [34] was used as a positive control. Lysate of the cells not exposed to rHP986 served as negative control.

### Statistical Analysis

Results were expressed as means ± the standard error (SE). Induction of cytokine levels and the rate of apoptosis were compared using a two-tailed Student's *t* test and considered significant if the *P* values were <0.05. *P* values were calculated using the online Graph Pad scientific calculator (http://www.graphpad.com_quickcalcs_ttest1.cfm).
